# Health and economic impacts of Lassa vaccination campaigns in West Africa

**DOI:** 10.1038/s41591-024-03232-y

**Published:** 2024-08-28

**Authors:** David R. M. Smith, Joanne Turner, Patrick Fahr, Lauren A. Attfield, Paul R. Bessell, Christl A. Donnelly, Rory Gibb, Kate E. Jones, David W. Redding, Danny Asogun, Oladele Oluwafemi Ayodeji, Benedict N. Azuogu, William A. Fischer, Kamji Jan, Adebola T. Olayinka, David A. Wohl, Andrew A. Torkelson, Katelyn A. Dinkel, Emily J. Nixon, Koen B. Pouwels, T. Déirdre Hollingsworth

**Affiliations:** 1 Nuffield Department of Population Health, Health Economics Research Centre, University of Oxford, Oxford, UK; 2 Department of Mathematical Sciences, University of Liverpool, Liverpool, UK; 3 Department of Livestock and One Health, Institute of Infection, Veterinary and Ecological Sciences, University of Liverpool, Liverpool, UK; 4 Nuffield Department of Primary Care Health Sciences, University of Oxford, Oxford, UK; 5 Department of Genetics, Evolution and Environment, Centre for Biodiversity and Environment Research, University College London, London, UK; 6 Department of Infectious Disease Epidemiology, Imperial College London, London, UK; 7 Independent consultant, Edinburgh, UK; 8 Department of Statistics, University of Oxford, Oxford, UK; 9 Pandemic Sciences Institute, University of Oxford, Oxford, UK; 10 Science Department, The Natural History Museum, London, UK; 11 Irrua Specialist Teaching Hospital, Irrua, Nigeria; 12 Federal Medical Centre, Owo, Nigeria; 13 Alex Ekwueme Federal University Teaching Hospital Abakaliki, Abakaliki, Nigeria; 14 Institute for Global Health and Infectious Diseases, University of North Carolina at Chapel Hill School of Medicine, Chapel Hill, NC USA; 15 Nigeria Centre for Disease Control and Prevention, Abuja, Nigeria; 16 Linksbridge SPC, Seattle, WA USA; 17 Big Data Institute, Li Ka Shing Centre for Health Information and Discovery, University of Oxford, Oxford, UK; 18 Nuffield Department of Medicine, NDM Centre for Global Health Research, University of Oxford, Oxford, UK

**Keywords:** Viral infection, Epidemiology, Outcomes research, Epidemiology, Health care economics

## Abstract

Lassa fever is a zoonotic disease identified by the World Health Organization (WHO) as having pandemic potential. This study estimates the health-economic burden of Lassa fever throughout West Africa and projects impacts of a series of vaccination campaigns. We also model the emergence of ‘Lassa-X’—a hypothetical pandemic Lassa virus variant—and project impacts of achieving 100 Days Mission vaccination targets. Our model predicted 2.7 million (95% uncertainty interval: 2.1–3.4 million) Lassa virus infections annually, resulting over 10 years in 2.0 million (793,800–3.9 million) disability-adjusted life years (DALYs). The most effective vaccination strategy was a population-wide preventive campaign primarily targeting WHO-classified ‘endemic’ districts. Under conservative vaccine efficacy assumptions, this campaign averted $20.1 million ($8.2–$39.0 million) in lost DALY value and $128.2 million ($67.2–$231.9 million) in societal costs (2021 international dollars ($)). Reactive vaccination in response to local outbreaks averted just one-tenth the health-economic burden of preventive campaigns. In the event of Lassa-X emerging, spreading throughout West Africa and causing approximately 1.2 million DALYs within 2 years, 100 Days Mission vaccination averted 22% of DALYs given a vaccine 70% effective against disease and 74% of DALYs given a vaccine 70% effective against both infection and disease. These findings suggest how vaccination could alleviate Lassa fever’s burden and assist in pandemic preparedness.

## Abstract (web summary)

A modeling study quantifies the health-economic burden of Lassa virus infection across West Africa and projects impacts of a series of reactive and preventive vaccination campaigns against the disease, presenting substantial impacts in terms of averted disability-adjusted life years and healthcare cost reductions.

## Main

Lassa fever is a viral hemorrhagic disease endemic to West Africa, where infections are common but widely undetected. Lassa fever is caused by *Lassa mammarenavirus* (LASV), and several lines of evidence, including detailed genomic analyses, suggest that the vast majority of human LASV infections are caused by zoonotic transmission from the Natal multimammate mouse (*Mastomys natalensis*)^1,2^. The virus can also spread through human-to-human contact, although this has predominantly been observed in healthcare settings with inadequate infection prevention and control practices^3^.

Most LASV infections are thought to be asymptomatic or cause only mild febrile illness^4^, but Lassa fever nonetheless has a large negative impact on population health and economies. Among patients presenting to hospital, the case–fatality ratio is estimated to be approximately 15%, and long-term sequelae, such as bilateral sensorineural hearing loss, are common in survivors of Lassa fever^5,6^. Monetary costs per hospitalization are estimated to be high and are often paid (partly) out of pocket by patients^7^. For example, a study from Nigeria found that the average patient’s out-of-pocket expenditure on Lassa fever treatment was approximately 480% of the monthly minimum wage in 2011 (ref. ^8^).

No licensed vaccines against Lassa fever are currently available, although several candidates are under development. A recent phase 1 randomized trial of a measles-vectored Lassa vaccine showed an acceptable safety and tolerability profile, a substantial increase in LASV-specific non-neutralizing IgG concentrations and a moderate T cell response^9^, in line with the response observed in non-human primates^10^. Several other vaccines are currently at early stages of development, with five phase 1 trials and one phase 2 trial registered by October 2022 (ref. ^11^).

Lassa fever is listed by the World Health Organization (WHO) as one of the diseases posing the greatest risk to public health due to its epidemic potential and the absence of effective countermeasures^12^. In response to such concerns, in 2022, the Group of Seven forum, the Group of Twenty forum and various international governments endorsed the 100 Days Mission, a pandemic response roadmap aiming at the delivery of vaccines within 100 d of the emergence of novel pathogens with pandemic potential^13^.

In anticipation of one or more Lassa vaccine candidates being licensed in the near future, in the present study, we estimate the current health-economic burden of Lassa fever in West Africa and project the potential impacts of different reactive and preventive vaccination campaigns. We also project potential impacts of vaccination in line with the 100 Days Mission in response to the emergence of ‘Lassa-X’, a hypothetical future variant of LASV with pandemic potential. Table 1 summarizes our main findings and their implications for public policy.

**Table 1:** Policy summary

Background	Lassa fever is a widely underreported emerging zoonotic disease caused by LASV, a priority pathogen identified by the WHO as having pandemic potential. At least four Lassa vaccine candidates are currently undergoing assessment in clinical trials. Here, we used a bottom-up mathematical modeling approach to provide the first estimates of Lassa fever’s health-economic burden across the 15 countries of continental West Africa and to project impacts of large-scale Lassa vaccination campaigns on population health and economies. We also modeled the emergence of ‘Lassa-X’, a hypothetical Lassa-related virus with increased transmissibility and virulence, and projected impacts of reactive vaccination in line with the goals of the 100 Days Mission.
Main findings and limitations	Over a 10-year horizon, our model estimated approximately 237,000 hospitalizations, 39,000 deaths and 2.0 million DALYs due to Lassa fever, totaling $506 million in direct healthcare costs, $1.1 billion in productivity losses and $288 million in monetized DALY value, or $15.3 billion in lost VSL. Large-scale preventive vaccination campaigns were more efficient than reactive outbreak response vaccination, requiring more doses but achieving greater health-economic benefit per dose. The most expansive campaign consisted mostly of preventive vaccination in WHO-classified endemic districts, requiring 112 million doses over 10 years and averting 20,000–29,000 hospitalizations (range of means across vaccine effectiveness assumptions), 3,300–4,800 deaths and 164,000–240,000 DALYs. In turn, this campaign averted $42–$61 million in healthcare costs, $86–$126 million in productivity losses and $20–$30 million in monetized DALY value, or $1.3–$1.9 billion in lost VSL. Prospective, population-based studies of Lassa fever epidemiology are limited, particularly outside of known Lassa fever hotspots. Our model-based analysis is, thus, sensitive to potential biases in available data inputs and does not explicitly estimate burden in high-risk groups, such as healthcare workers and pregnant women. This impedes evaluation of risk-targeted vaccination campaigns. In the hypothetical event of Lassa-X emerging, our modeling suggests that its rapid spread throughout West Africa could result in 25,000 deaths and 1.2 million DALYs within approximately 2 years. For a vaccine 70% effective against both infection and disease, mass reactive vaccination beginning 100 d from the disease’s discovery averted 11% of DALYs at a vaccination rate of 2.5% of the population annually (10 million doses per year), 55% of DALYs at 20% (81 million) and 74% of DALYs at 40% (161 million).
Policy implications	In endemic regions of West Africa, the estimated DALY burden of Lassa fever is similar to estimates for other infectious diseases, such as rabies, lymphatic filariasis and intestinal nematode infections. Population-wide preventive vaccination campaigns in endemic regions could substantially reduce the health and economic burden of disease. Our model suggests the potential for substantial added benefit to expanding vaccination beyond WHO-classified endemic districts. However, prospective cohort studies are needed to better define groups at high risk of infection and severe disease, in turn informing potential risk-targeted immunization strategies, which may be more cost-effective than the population-wide campaigns considered in our analysis. Investment in Lassa vaccine development and infrastructure could also improve preparedness for the rapid development of new vaccines for potential future Lassa-related viruses. In the event of a hypothetical pandemic variant of Lassa virus emerging, achieving 100 Days Mission vaccination goals was projected to yield critical health and economic benefits, including the aversion of up to three-quarters of associated DALYs. Vaccine effectiveness against infection in addition to disease would be essential to slow spread and mitigate such an emergent pathogen’s large-scale health and economic toll.

Monetary costs are reported in 2021 international dollars ($).

## Results

### Model overview

We developed an epidemiological model projecting human Lassa fever burden over a 10-year time horizon across the 15 countries of continental West Africa (Benin, Burkina Faso, Côte d’Ivoire, The Gambia, Ghana, Guinea, Guinea-Bissau, Liberia, Mali, Mauritania, Niger, Nigeria, Senegal, Sierra Leone and Togo) and their 183 level 1 subnational administrative units. These units have different names in different countries (for example, regions in Guinea, counties in Liberia and departments in Benin) but herein are collectively referred to as ‘districts’. Due to large gaps in Lassa fever surveillance and limited case reporting throughout much of its endemic range^3^, we favored a bottom-up modeling approach, synthesizing best available ecological, epidemiological, clinical and economic data to project the cumulative health and economic burden of disease.

**Extended Data Fig. 1:**
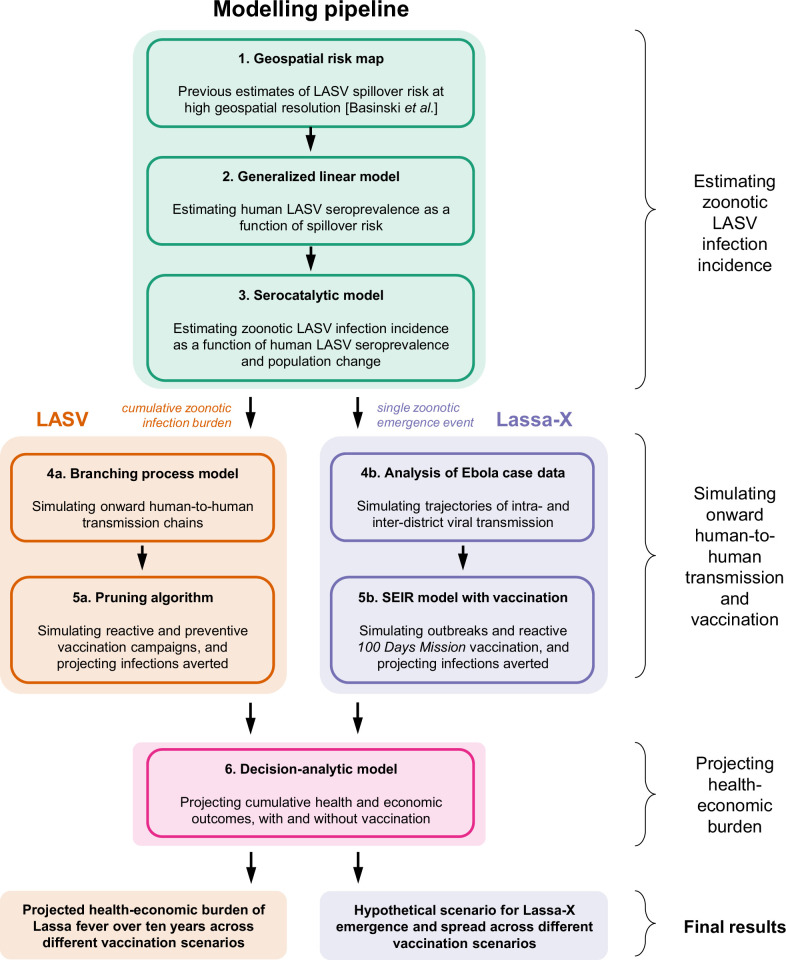
Model schematic. See Methods for details and Supplementary figure D.1 for a schematic of the decision-analytic model describing disease progression. Pruning in step 5a refers to retrospectively removing infections averted due to vaccination from simulated transmission chains. LASV = *Lassa virus*.

Our model consists of six main components (see model schematic in Extended Data Fig. 1). First, a previously published geospatial risk map was used to predict the risk of zoonotic LASV transmission from *M. natalensis* to humans (‘spillover’) at the level of 0.05° × 0.05° spatial pixels throughout West Africa^14^. Second, modeled spillover risk estimates were used as inputs in a generalized linear model (GLM) to predict human LASV seroprevalence. Third, modeled human LASV seroprevalence estimates were used as inputs in a serocatalytic model including country-level population projections to predict spillover infection incidence. Fourth, spillover infections were aggregated at the district level, and a stochastic branching process model was used to simulate onward human-to-human LASV transmission. Fifth, a computational algorithm was applied retrospectively to spillover infections and ensuing transmission chains to simulate a range of reactive and preventive vaccination campaigns and to project the number of infections averted by vaccination. (Separate model components used to simulate Lassa-X transmission and vaccination are described below.) Sixth, modeled estimates of LASV infection, and of infections averted due to vaccination or occurring in vaccinated individuals, were used as inputs in a probabilistic decision-analytic model used to project the health burden of Lassa fever and associated economic costs and the health and economic burden averted due to vaccination over 10 years.

### Lassa fever burden

**Fig. 1:**
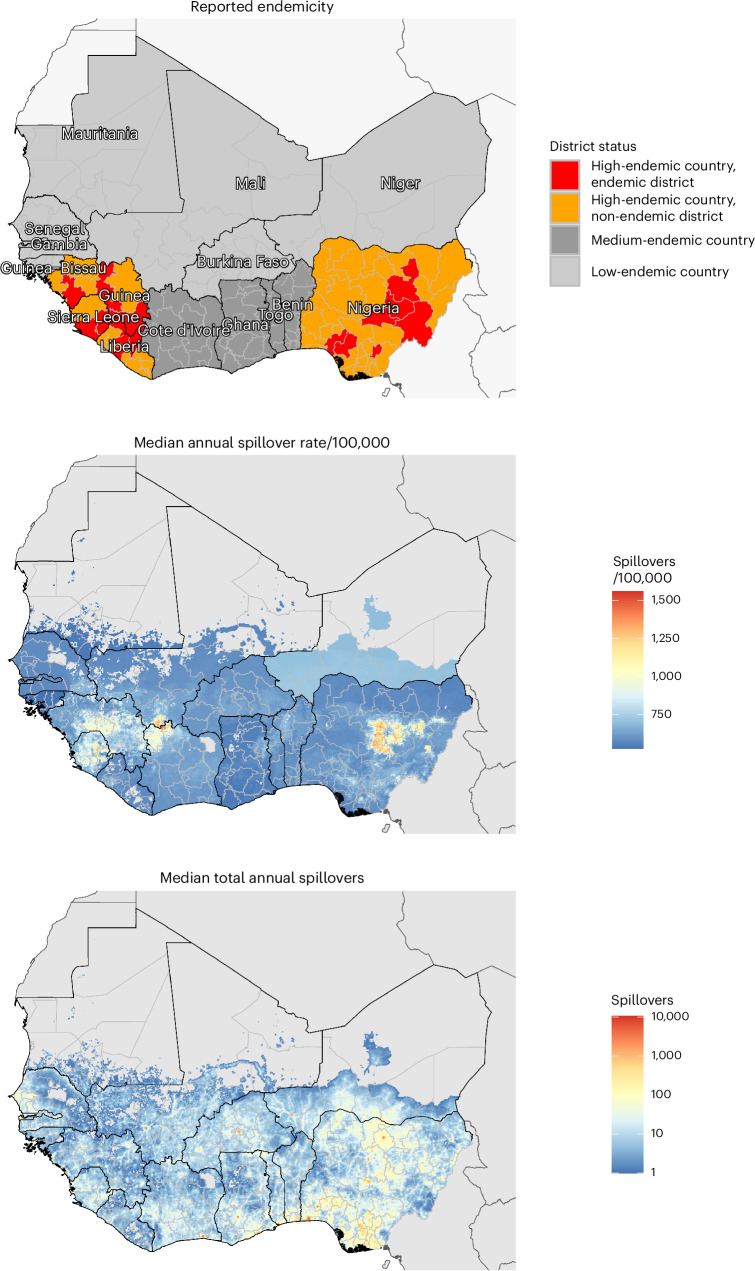
Maps of West Africa showing reported Lassa fever endemicity and estimated LASV spillover incidence. Top, map showing the classification of Lassa fever endemicity for different countries and ‘districts’, as defined by the US CDC and the WHO (Supplementary Appendix C.2). Middle, the median annual incidence of zoonotic LASV infection per 100,000 population as estimated by our model at the level of 5-km grid cells. Bottom, the median total annual number of zoonotic LASV infections as estimated by our model at the level of 5-km grid cells.

Our model predicts a heterogeneous distribution of zoonotic LASV infection throughout West Africa (Fig. 1). In the absence of vaccination, the mean annual number of LASV infections throughout the region was estimated at 2.7 million (95% uncertainty interval (UI): 2.1–3.4 million) or 27.2 million (20.9–34.0 million) over the full 10-year simulation period (Extended Data Table 1). Just over half of all infections occurred in Nigeria (mean, 52.9%), and the vast majority (mean, 93.7%) resulted from zoonotic spillover as opposed to human-to-human transmission, due to LASV’s low estimated basic reproduction number (*R*_0_). At the district level, annual LASV infection incidence was highest in Margibi, Liberia (1,198 (943–1,475) infections per 100,000 population), followed by Denguélé, Côte d’Ivoire (1,032 (880–1,200) per 100,000 population) and Nasarawa, Nigeria (978 (803–1,162) per 100,000 population). Over 10 years, LASV infection throughout West Africa led to an estimated 5.4 million (2.7–9.9 million) mild/moderate symptomatic cases, 237,000 (148,600–345,600) hospitalizations and 39,300 (12,900–83,300) deaths, resulting in 2.0 million (793,800–3.9 million) disability-adjusted life years (DALYs). See Supplementary Appendix E for more detailed estimates of Lassa fever burden.

**Extended Data Fig. 2:**
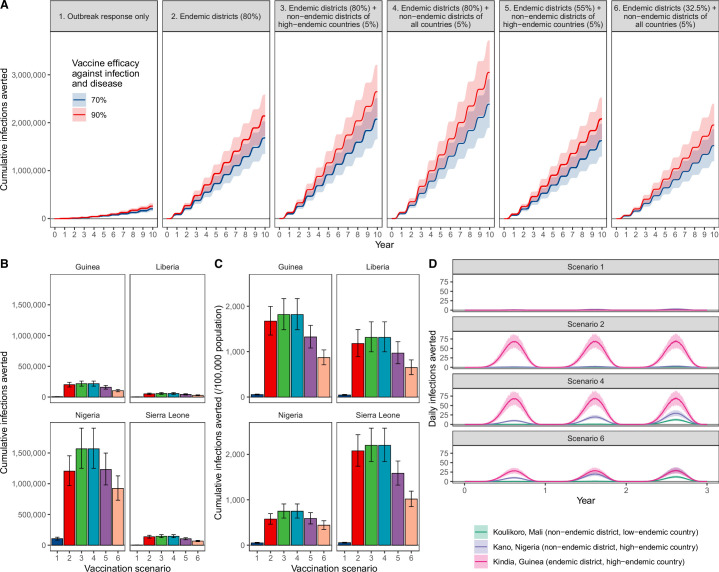
Impacts of a Lassa vaccine effective against infection and disease. (**A**) The mean cumulative number of LASV infections averted due to vaccination across the 15 countries included in the model, comparing vaccine efficacy against infection and disease of 70% (blue) versus 90% (red) across the six considered vaccination scenarios (panels). 95% uncertainty intervals are indicated by shading. (**B**) The mean cumulative number of infections averted over ten years under each vaccination scenario in the four countries classified as high-endemic (Guinea, Liberia, Nigeria and Sierra Leone). 95% uncertainty intervals are indicated by error bars (n = 9,900). (**C**) The mean cumulative incidence of infections averted over ten years per 100,000 population under each vaccination scenario in the same four countries. 95% uncertainty intervals are indicated by error bars (n = 9,900). (**D**) The mean daily number of infections averted by a vaccine with 70% efficacy against infection and disease over the first three years of vaccine rollout, in three distinct districts under four selected vaccination scenarios. 95% uncertainty intervals are indicated by shading.

Over 10 years, Lassa fever treatment was projected to incur $338.9 million ($206.6–$506.3 million) in government-reimbursed treatment costs and $166.9 million ($116.0–$289.3 million) in out-of-pocket medical costs, resulting in catastrophic expenditures for 232,300 (145,600–338,700) individuals and pushing 167,000 (104,700–243,600) individuals below the international poverty line (Supplementary Tables E.3 and E.4). Missed work due to illness totaled $1.1 billion ($380.5 million–$2.2 billion) in productivity losses, primarily due to mortality in actively employed adults. Productivity losses outranked treatment costs in driving an estimated $1.6 billion ($805.1 million–$2.8 billion) in total cumulative societal costs. Hospitalization costs, not outpatient costs, were the main driver of treatment costs, but mild to moderate disease in the community resulted in greater productivity losses than severe disease in hospital (Supplementary Fig. E.2). Lassa fever DALYs were valued at $287.7 million ($115.4–$562.9 million) using country-specific cost-effectiveness thresholds. Finally, an alternative measure of Lassa fever’s economic burden, the value of statistical life (VSL) lost due to Lassa fever mortality, was projected at $15.3 billion ($5.0–$32.4 billion). Uncertainty in health-economic outcomes was primarily driven by uncertainty in risks of hospitalization and death (Supplementary Fig. D.2)

### Simulating Lassa vaccination campaigns

Vaccination is introduced into the population via a series of six scenarios designed to reflect realistic assumptions about vaccine stockpile, administration and efficacy (Extended Data Table 2). In all six scenarios, we include reactive vaccination, in which Lassa fever outbreaks trigger the local deployment of a limited vaccine stockpile in affected districts. In scenarios 2–6, we also include preventive vaccination in the form of mass, population-wide campaigns rolled out over 3 years and focusing primarily on regions classified as Lassa fever ‘endemic’. The 15 countries included in our model are categorized as high endemic, medium endemic or low endemic according to classifications published by the US Centers for Disease Control and Prevention (CDC), and districts within high-endemic countries are further classified as endemic or non-endemic according to classifications published by the WHO (Fig. 1 and Supplementary Appendix C.2). Two main mechanisms of vaccine efficacy are considered: protection against infection prevents individuals from acquiring LASV infection from either *M. natalensis* or other humans, and protection against disease prevents vaccinated individuals who become infected from progressing to disease, thus averting outpatient consultation, hospitalization, chronic sequelae and death. In our simulations, we project impacts of a vaccine that is 70% or 90% effective only against disease or 70% or 90% effective against both infection and disease. We do not consider other potential mechanistic impacts of vaccination, such as reduced infectiousness or altered behavior among vaccinated individuals, as such factors are less relevant given low estimated rates of human-to-human LASV transmission.

### Health-economic impacts of vaccination against Lassa fever

The considered vaccination scenarios varied considerably in their projected impacts, with scenario 4 leading to the greatest reductions in Lassa fever burden over 10 years (Extended Data Fig. 2 and Table 2). In this scenario, in addition to reactive vaccination triggered in districts experiencing local outbreaks, preventive vaccination was administered to 80% of the population in WHO-classified endemic districts as well as to 5% of the population in all other districts throughout West Africa. For a vaccine 70% effective against disease with no impact on infection, over 10 years this strategy averted a mean 456,000 (226,400–822,700) mild/moderate symptomatic cases, 19,900 (12,700–28,800) hospitalizations, 3,300 (1,100–7,000) deaths and 164,100 (66,700–317,700) DALYs. Over this period, this strategy further prevented 19,800 (12,600–28,500) and 14,200 (9,000–20,500) individuals, respectively, from experiencing catastrophic or impoverishing out-of-pocket healthcare expenditures and averted $128.2 million ($67.2–$231.9 million) in societal costs, or $1.3 billion ($436.8 million–$2.8 billion) in VSL lost.

**Table 2:** Projected 10-year impacts of Lassa vaccination

Outcome averted due to vaccination	Scenario 1: outbreak response only	Scenario 2: endemic districts (80%)	Scenario 3: endemic districts (80%) + non-endemic districts of high-endemic countries (5%)	Scenario 4: endemic districts (80%) + non-endemic districts of all countries (5%)	Scenario 5: endemic districts (55%) + non-endemic districts of high-endemic countries (5%)	Scenario 6: endemic districts (32.5%) + non-endemic districts of all countries (5%)
**Vaccine 70% effective only against disease**						
LASV infections (*n*)	0.0 (0.0–0.0)	0.0 (0.0–0.0)	0.0 (0.0–0.0)	0.0 (0.0–0.0)	0.0 (0.0–0.0)	0.0 (0.0–0.0)
Hospitalizations (*n*)	1.6 K (1.0 K–2.4 K)	14.1 K (9.0 K–20.3 K)	17.3 K (11.1 K–25.0 K)	19.9 K (12.7 K–28.8 K)	13.4 K (8.6 K–19.4 K)	12.5 K (7.9 K–18.1 K)
Deaths (*n*)	272.0 (89.0–576.8)	2.3 K (771.7–4.9 K)	2.9 K (947.3–6.0 K)	3.3 K (1.1 K–7.0 K)	2.2 K (734.0–4.7 K)	2.1 K (680.7–4.4 K)
DALYs (*n*)	13.7 K (5.5 K–26.8 K)	115.4 K (47.1 K–222.2 K)	141.4 K (57.6 K–273.2 K)	164.1 K (66.7 K–317.7 K)	109.6 K (44.6 K–211.9 K)	103.8 K (41.9 K–201.4 K)
Impoverishing expenditures (*n*)	1.2 K (725.0–1.7 K)	10.1 K (6.5 K–14.5 K)	12.4 K (7.9 K–17.8 K)	14.2 K (9.0 K–20.5 K)	9.6 K (6.1 K–13.8 K)	8.9 K (5.6 K–12.9 K)
Societal costs (2021 $)	10.3 M (5.3 M–18.8 M)	90.3 M (47.6 M–162.5 M)	112.8 M (59.2 M–203.8 M)	128.2 M (67.2 M–231.9 M)	87.9 M (46.0 M–158.9 M)	80.8 M (42.1 M–146.6 M)
Monetized DALYs (2021 $)	1.9 M (763.1 K–3.7 M)	12.9 M (5.3 M–24.9 M)	15.8 M (6.4 M–30.4 M)	20.1 M (8.2 M–39.0 M)	12.3 M (5.0 M–23.8 M)	13.6 M (5.5 M–26.4 M)
VSL (2021 $)	105.7 M (34.6 M–224.1 M)	948.9 M (313.2 M–2.0 B)	1.2 B (396.6 M–2.5 B)	1.3 B (436.8 M–2.8 B)	939.7 M (309.4 M–2.0 B)	826.3 M (271.1 M–1.7 B)
**Vaccine 70% effective against infection and disease**						
LASV infections (*n*)	200.2 K (153.5 K–250.0 K)	1.7 M (1.3 M–2.0 M)	2.1 M (1.6 M–2.5 M)	2.4 M (1.9 M–2.9 M)	1.6 M (1.3 M–2.0 M)	1.5 M (1.2 M–1.9 M)
Hospitalizations (*n*)	2.2 K (1.4 K–3.3 K)	18.8 K (12.0 K–27.1 K)	23.2 K (14.8 K–33.5 K)	26.8 K (17.0 K–38.6 K)	18.1 K (11.6 K–26.2 K)	17.0 K (10.8 K–24.6 K)
Deaths (*n*)	370.6 (121.3–785.9)	3.1 K (1.0 K–6.6 K)	3.9 K (1.3 K–8.1 K)	4.4 K (1.5 K–9.4 K)	3.0 K (990.8–6.3 K)	2.8 K (924.5–6.0 K)
DALYs (*n*)	18.7 K (7.5 K–36.5 K)	154.2 K (63.0 K–296.8 K)	189.6 K (77.3 K–366.3 K)	220.5 K (89.6 K–427.0 K)	148.0 K (60.2 K–286.1 K)	140.9 K (56.9 K–273.6 K)
Impoverishing expenditures (*n*)	1.6 K (987.8–2.3 K)	13.5 K (8.6 K–19.4 K)	16.6 K (10.6 K–23.9 K)	19.1 K (12.1 K–27.6 K)	12.9 K (8.2 K–18.6 K)	12.1 K (7.6 K–17.5 K)
Societal costs (2021 $)	14.0 M (7.3 M–25.6 M)	120.6 M (63.7 M–217.1 M)	151.4 M (79.4 M–273.5 M)	172.3 M (90.3 M–311.7 M)	118.6 M (62.1 M–214.6 M)	109.7 M (57.1 M–199.2 M)
Monetized DALYs (2021 $)	2.6 M (1.0 M–5.1 M)	17.3 M (7.1 M–33.3 M)	21.1 M (8.6 M–40.8 M)	27.1 M (11.0 M–52.5 M)	16.6 M (6.8 M–32.2 M)	18.4 M (7.4 M–35.9 M)
VSL (2021 $)	144.1 M (47.2 M–305.4 M)	1.3 B (418.5 M–2.7B)	1.6 B (532.1 M–3.4 B)	1.8 B (586.7 M–3.8 B)	1.3 B (417.8 M–2.7 B)	1.1 B (368.1 M–2.4 B)

The mean (95% UI) health and economic burden of Lassa fever averted due to vaccination over 10 years from the initiation of vaccine rollout for the six vaccination scenarios described in Extended Data Table 2. Columns represent vaccination scenarios, and rows represent outcomes averted. The table compares a vaccine 70% effective only against disease (top) with a vaccine 70% effective against both infection and disease (bottom). Societal costs combine outpatient treatment costs, hospital treatment costs and productivity losses. Costs are reported in 2021 international dollars ($), and future monetary costs are discounted at 3% per year. B, billion; K, thousand; M, million.

Other vaccination scenarios used fewer doses of vaccine and, in turn, averted less of Lassa fever’s health-economic burden. Scenario 3, which limited preventive vaccination to high-endemic countries, was the scenario resulting in the second greatest health-economic benefits, including the aversion of 141,400 (57,600–273,200) DALYs and $112.8 million ($59.2–$203.8 million) in societal costs. Scenarios 2, 5 and 6 varied considerably in terms of which individuals were vaccinated but ultimately resulted in similar cumulative health-economic benefits across the region, because the overall number of doses delivered under each scenario was essentially the same. By contrast, scenario 1 included only reactive and not preventive vaccination, averting just 13,700 (5,500–26,800) DALYs and $10.3 million ($5.3–$18.8 million) in societal costs, thus having approximately one-tenth the overall health-economic benefits of scenario 4.

A vaccine effective against infection in addition to disease was found to have moderately increased impact. In scenario 4, for instance, $20.1 million ($8.2–$39.0 million) in DALY value was averted by a vaccine 70% effective only against disease, whereas $27.1 million ($11.0–$52.5 million) was averted when also 70% effective against infection (Table 2). By comparison, a vaccine 90% effective only against disease averted $25.8 million ($10.5–$50.1 million) in DALY value (Supplementary Table E.9), having similar impact to a vaccine 70% effective against both infection and disease. In the best-case scenario of a vaccine 90% effective against both infection and disease, scenario 4 averted up to 3.1 million (2.4–3.7 million) infections, 240,100 (97,500–464,900) DALYs valued at $29.5 million ($12.0–$57.2 million) and $1.9 billion ($638.5 million–$4.1 billion) in VSL lost.

**Fig. 2:**
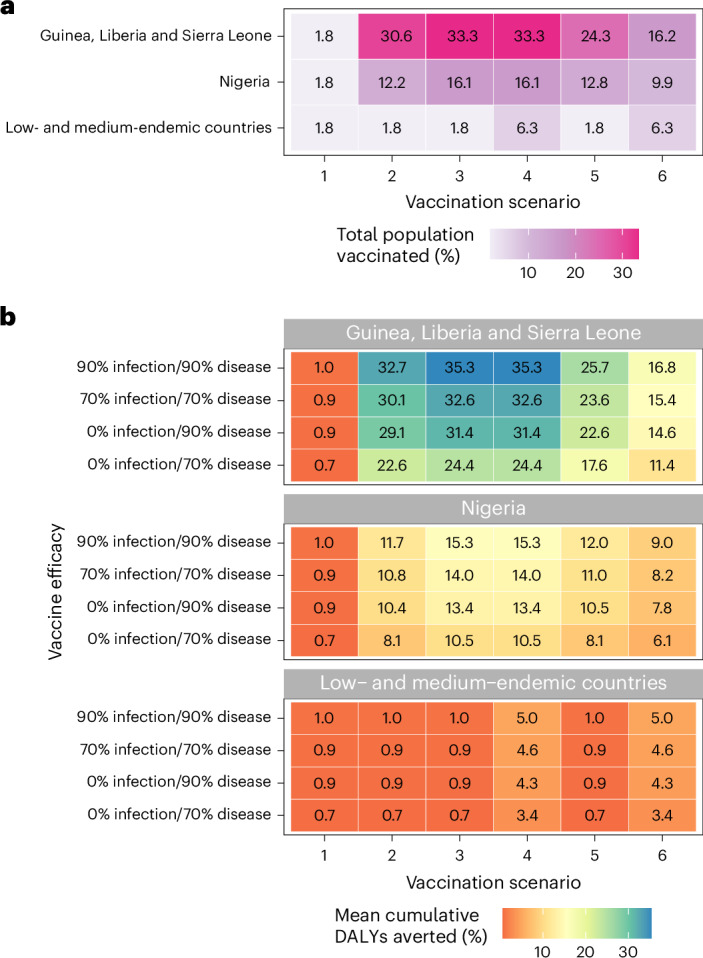
Vaccination coverage and corresponding reductions in Lassa fever burden vary greatly across countries. **a**, Share of the total population vaccinated by 10 years in each vaccination scenario (*x* axis) and aggregated across three geographic levels (*y* axis). **b**, Share of cumulative DALYs due to Lassa fever averted over 10 years by vaccination. Impacts vary greatly depending on the vaccination scenario (*x* axis), the assumed vaccine efficacy (*y* axis) and the geographic location (panels).

Geographic variation in vaccine impact depended primarily on which districts were classified as endemic and, hence, targeted for vaccination (Extended Data Fig. 2). Overall impacts of vaccination were greatest in Nigeria, but impacts per 100,000 population were greatest in other endemic countries (Guinea, Liberia and Sierra Leone), because Nigeria had a larger number of individuals but a smaller share of its total population living in districts classified as endemic. In turn, approximately 16% of the total population of Nigeria and 33% of the combined population of Guinea, Liberia and Sierra Leone were vaccinated by 10 years under scenarios 3 and 4 (Fig. 2). Given a vaccine 70% effective only against disease, these scenarios averted 10.5% of DALYs in Nigeria, 20.3% of DALYs in Liberia, 23.6% of DALYs in Guinea and 28.1% of DALYs in Sierra Leone. For a vaccine 90% effective against infection and disease, these scenarios averted 15.3% of DALYs in Nigeria, 29.4% of DALYs in Liberia, 34.1% of DALYs in Guinea and 40.7% of DALYs in Sierra Leone.

### Threshold vaccine costs

Projected economic benefits of Lassa vaccination were used to calculate the threshold vaccine cost (TVC). This can be interpreted as the maximum cost per dose at which vaccination has a benefit-to-cost ratio above 1, in the specific context of our modeled vaccination campaigns and corresponding dosage assumptions (that is, a single-dose primary series followed by a single-dose booster after 5 years, with 10% dose wastage). TVCs were similar across all five preventive campaigns (scenarios 2–6) but lower for reactive vaccination (scenario 1) (Supplementary Table E.12). Estimated TVCs ranged from $0.51 ($0.30–$0.80) to $21.15 ($7.28–$43.97) depending on the economic perspective considered, the vaccination campaign evaluated and the vaccine’s efficacy against infection and disease. TVCs were lowest from the perspective considering only healthcare costs and monetized DALYs (range of means, $0.51–$0.91) but more than doubled given a perspective considering all societal costs (healthcare costs and productivity losses) in addition to monetized DALYs ($1.18–$2.20) and increased by more than 20-fold when considering healthcare costs and VSL ($10.54–$21.15).

### Modeling ‘Lassa-X’

In addition to our analysis of Lassa fever, we modeled the emergence of ‘Lassa-X’, a hypothetical future variant of LASV with pandemic potential due to both elevated clinical severity and increased propensity for human-to-human transmission. In this analysis, Lassa-X was assumed to emerge in humans after a single spillover event, where the probability of emergence in each district is directly proportional to the estimated share of all zoonotic LASV infections occurring in each district. We assumed that prior LASV immunity, whether natural or vaccine derived, offers no protection against Lassa-X. We conceptualized Lassa-X as having Ebola-like transmission characteristics and, under baseline assumptions, a 10-fold increase in hospitalization risk relative to Lassa fever. Lassa-X transmission parameters were quantified using Ebola case data from the 2013/2016 West Africa epidemic, resulting in simulated Lassa-X outbreaks lasting for approximately 2 years before subsiding. A range of reactive 100 Days Mission vaccination scenarios were then evaluated, considering different delays to vaccine initiation, rates of vaccine uptake and degrees of efficacy against infection and disease. Finally, as for Lassa fever, we used a probabilistic decision-analytic model to project the health and economic burden of Lassa-X and burden averted as a result of vaccination.

### Projected burden of Lassa-X

**Fig. 3:**
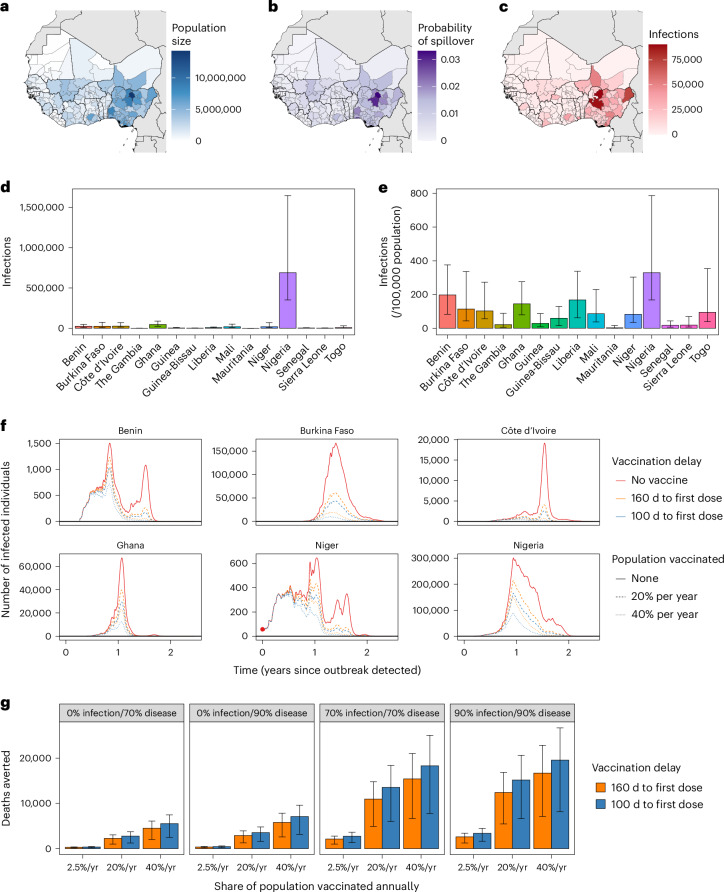
Projected burden of Lassa-X infection and impacts of vaccination. **a**–**c**, Maps of West Africa showing, for each district: the population size (**a**), the probability of Lassa-X spillover (**b**) and the mean cumulative number of Lassa-X infections over the entire outbreak (approximately 2 years) (**c**). **d**,**e**, The second row depicts the median cumulative incidence of Lassa-X infection over the entire outbreak (**d**) and the median cumulative incidence over the entire outbreak per 100,000 population in the absence of vaccination (**e**). Interquartile ranges are indicated by error bars (*n* = 10,000). **f**, The total number of Lassa-X infections over time in six selected countries in one randomly selected outbreak simulation in which the initial Lassa-X spillover event occurred in Niger (the red dot highlights the initial detection of the epidemic at time 0). Lines show how a vaccine with 70% efficacy against infection and disease influences infection dynamics, where line color represents the delay to vaccine rollout, and line dashing represents the rate of vaccination (the proportion of the population vaccinated over a 1-year period). **g**, The mean cumulative number of deaths averted due to vaccination over the entire outbreak and across all countries, depending on vaccine efficacy (panels), the rate of vaccination (*x* axis) and the delay to vaccine rollout (colors). Interquartile ranges are indicated by error bars (*n* = 10,000). yr, year.

Under our modeling assumptions, the emergence of Lassa-X led to explosive outbreaks throughout West Africa (Fig. 3), spreading to 88.3% (63.9%–94.0%) of the 183 districts included in our model (Supplementary Fig. F.1). In total, there were 1.7 million (230,100–4.2 million) Lassa-X infections, and Nigeria accounted for by far the greatest share of infections, followed by Niger and Ghana (Supplementary Tables G.1 and G.2). The projected burden of Lassa-X infection was associated with a high degree of uncertainty, driven predominantly by the highly stochastic nature of simulated outbreaks (Supplementary Fig. G.2).

In our baseline analysis, Lassa-X resulted in 149,700 (19,700–374,400) hospitalizations and 24,800 (2,400–76,000) deaths, causing 1.2 million (132,500–3.7 million) DALYs valued at $191.1 million ($18.4–$575.2 million). Out-of-pocket treatment costs were estimated at $118.5 million ($12.2–$317.3 million), resulting in catastrophic healthcare expenditures for 147,400 (18,500–372,500) individuals and pushing 103,100 (13,600–254,300) individuals below the poverty line. Lassa-X also resulted in $737.2 million ($56.4 million–$2.4 billion) in productivity losses to the greater economy and $10.1 billion ($625.9 million–$34.1 billion) in VSL lost. In alternative scenarios where Lassa-X infection was just as likely or one-tenth as likely to result in hospitalization as LASV infection, estimates of the health-economic burden were approximately one and two orders of magnitude lower, respectively (Supplementary Table G.4).

### Vaccination to slow the spread of Lassa-X

Impacts of vaccination on the health-economic burden of Lassa-X depend on the delay until vaccination initiation, the rate of vaccine uptake in the population and the efficacy of vaccination against infection and/or disease (Table 3). In the most ambitious vaccination scenario considered, vaccine administration began 100 d after initial detection of the first hospitalized case of Lassa-X at a rate equivalent to 40% of the population per year across all countries in West Africa. Assuming a vaccine 70% effective only against disease, this vaccination scenario averted 276,600 (38,000–755,900) DALYs. However, in contrast to LASV vaccination, vaccine impact was more than three-fold greater when effective against infection as well as disease. For a vaccine 70% effective against both, this most ambitious vaccination scenario averted 1.2 million (201,300–2.7 million) infections and 916,400 (108,000–2.6 million) DALYs, representing approximately 74% of the DALY burden imposed by Lassa-X. Vaccinating at half the rate (20% of the population per year) averted approximately 55% of the DALYs imposed by Lassa-X, whereas vaccinating at a low rate (2.5% of the population per year) averted just 11% of DALYs (Supplementary Tables G.5–G.8). Benefits of delivering vaccines at a higher rate outweighed benefits of initiating vaccination earlier (100 d versus 160 d from outbreak detection), which, in turn, outweighed benefits of a vaccine with greater efficacy against infection and disease (90% versus 70%).

**Table 3:** Projected impacts of 100 Days Mission vaccination campaigns in response to Lassa-X

Outcome averted due to vaccination	Vaccination scenario					
	2.5% of population vaccinated per year		20% of population vaccinated per year		40% of population vaccinated per year	
	160-d delay to first dose	100-d delay to first dose	160-d delay to first dose	100-d delay to first dose	160-d delay to first dose	100-d delay to first dose
**Vaccine 70% effective only against disease**						
Lassa-X infections (*n*)	0.0 (0.0–0.0)	0.0 (0.0–0.0)	0.0 (0.0–0.0)	0.0 (0.0–0.0)	0.0 (0.0–0.0)	0.0 (0.0–0.0)
Hospitalizations (*n*)	1.7 K (294.0–3.9 K)	2.1 K (361.5–4.7 K)	13.6 K (2.4 K–31.2 K)	16.6 K (2.9 K–37.3 K)	27.1 K (4.7 K–62.3 K)	33.3 K (5.8 K–74.8 K)
Deaths (*n*)	281.2 (35.6–811.0)	344.5 (43.9–984.0)	2.2 K (284.6–6.5 K)	2.8 K (350.9–7.9 K)	4.5 K (569.2–13.0 K)	5.5 K (704.9–15.7 K)
DALYs (*n*)	14.1 K (1.9 K–39.2 K)	17.3 K (2.4 K–47.2 K)	112.9 K (15.4 K–313.3 K)	138.1 K (18.9 K–377.6 K)	225.9 K (30.9 K–626.2 K)	276.6 K (38.0 K–755.9 K)
Impoverishing expenditures (*n*)	1.2 K (206.2–2.7 K)	1.4 K (255.7–3.2 K)	9.3 K (1.6 K–21.3 K)	11.4 K (2.0 K–25.3 K)	18.7 K (3.3 K–42.7 K)	22.9 K (4.1 K–50.5 K)
Societal costs (2021 $)	11.9 M (1.8 M–31.9 M)	14.6 M (2.2 M–38.8 M)	95.0 M (14.3 M–255.1 M)	116.8 M (17.7 M–310.5 M)	190.0 M (28.6 M–510.0 M)	233.7 M (35.5 M–620.8 M)
Monetized DALYs (2021 $)	2.2 M (325.8 K–6.6 M)	2.7 M (398.8 K–7.9 M)	17.9 M (2.6 M–52.8 M)	21.8 M (3.2 M–63.2 M)	35.8 M (5.3 M–105.8 M)	43.6 M (6.4 M–126.5 M)
VSL (2021 $)	109.8 M (11.3 M–346.3 M)	135.7 M (13.8 M–420.9 M)	878.2 M (90.6 M–2.8 B)	1.1 B (110.3 M–3.4 B)	1.8 B (181.6 M–5.5 B)	2.2 B (221.7 M–6.7 B)
**Vaccine 70% effective against infection and disease**						
Lassa-X infections (*n*)	141.4 K (27.5 K–323.4 K)	183.8 K (37.3 K–399.2 K)	737.6 K (146.5 K–1.7 M)	916.0 K (189.7 K–2.0 M)	1.0 M (200.5 K–2.3 M)	1.2 M (201.3 K–2.7 M)
Hospitalizations (*n*)	12.8 K (2.4 K–31.3 K)	16.6 K (3.1 K–38.8 K)	66.1 K (11.6 K–152.2 K)	81.8 K (14.4 K–184.8 K)	93.0 K (15.4 K–214.5 K)	110.5 K (16.8 K–252.3 K)
Deaths (*n*)	2.1 K (288.7–6.3 K)	2.7 K (380.1–7.9 K)	11.0 K (1.4 K–31.4 K)	13.6 K (1.6 K–38.6 K)	15.4 K (1.8 K–44.6 K)	18.3 K (2.0 K–53.1 K)
DALYs (*n*)	106.7 K (15.6K–304.9 K)	138.0 K (20.6K–383.0 K)	550.4 K (74.1K–1.5 M)	679.1 K (90.0K–1.9 M)	773.2 K (97.8K–2.1 M)	916.4 K (108.0K–2.6 M)
Impoverishing expenditures (*n*)	8.8 K (1.7 K–21.9 K)	11.4 K (2.2 K–26.9 K)	45.5 K (8.1 K–105.0 K)	56.2 K (10.0 K–126.9 K)	63.8 K (10.5 K–147.3 K)	75.9 K (11.6 K–173.4 K)
Societal costs (2021 $)	88.8 M (14.4 M–244.5 M)	115.8 M (19.1 M–310.0 M)	464.4 M (69.3 M–1.3 B)	576.9 M (80.4 M–1.6 B)	656.0 M (85.6 M–1.8 B)	782.9 M (91.4 M–2.2 B)
Monetized DALYs (2021 $)	17.0 M (2.6 M–51.6 M)	21.9 M (3.5 M–64.4 M)	88.1 M (12.2 M–267.6 M)	107.9 M (13.9 M–321.8 M)	123.2 M (14.9 M–374.0 M)	144.5 M (16.1 M–429.3 M)
VSL (2021 $)	809.8 M (95.2 M–2.6 B)	1.1 B (126.4 M–3.3 B)	4.3 B (423.4 M–13.8 B)	5.4 B (482.0 M–16.9 B)	6.1 B (518.6 M–19.7 B)	7.3 B (557.3 M–23.5 B)

The health-economic burden of Lassa-X averted due to vaccination, comparing a vaccine 70% effective only against disease (top) with a vaccine 70% effective against both infection and disease (bottom). Columns represent the vaccination scenarios considered, and rows represent the outcomes averted. All figures represent means (95% UIs) across all simulations for the baseline scenario, assuming a 10-fold greater risk of hospitalization relative to Lassa virus infection. Societal costs combine outpatient treatment costs, hospital treatment costs and productivity losses. Costs are reported in 2021 international dollars ($), and future monetary costs are discounted at 3% per year. B, billion; K, thousand; M, million.

## Discussion

This is, to our knowledge, the first burden of disease study for Lassa fever and the first to project impacts of Lassa vaccination campaigns on population health and economies^15^. We estimated that 2.1–3.4 million human LASV infections occur annually throughout West Africa, resulting in 15,000–35,000 hospitalizations and 1,300–8,300 deaths. These figures are consistent with recent modeling work estimating 900,000–4.4 million human LASV infections per year^14^ and an annual 5,000 deaths reported elsewhere^3,16^. We further estimated that Lassa fever causes 2.0 million DALYs, $1.6 billion in societal costs and $15.3 billion in lost VSL over 10 years. Our modeling suggests that administering Lassa vaccines preventively to districts of Nigeria, Guinea, Liberia and Sierra Leone that are currently classified as ‘endemic’ by the WHO would avert a substantial share of the burden of disease in those areas. In our most expansive rollout scenario, in which vaccine reaches approximately 80% of individuals in endemic districts and 5% of individuals elsewhere over a 3-year period, a vaccine 70% effective against disease is projected to avert 164,000 DALYs, $128 million in societal costs and $1.3 billion in VSL lost over 10 years. This corresponds to a 10.5% reduction in Lassa fever DALYs in Nigeria given vaccination among 16.1% of the population and a 24.4% reduction in DALYs across Guinea, Liberia and Sierra Leone given vaccination among 33.3% of the population. However, for the same rollout scenario, a vaccine 90% effective against both infection and disease could avert 240,000 DALYs, $188 million in societal costs and $1.9 billion in VSL lost, corresponding to a 15.3% reduction in Lassa fever DALYs in Nigeria and a 35.3% reduction across Guinea, Liberia and Sierra Leone.

Impacts of the Lassa vaccination campaigns included in our analysis were modest in countries other than Nigeria, Guinea, Liberia and Sierra Leone. This is due primarily to these simulated campaigns reflecting a constrained global vaccine stockpile (<20 million doses annually) and, hence, limited allocation to districts not currently classified as endemic by the WHO. Although our most optimistic vaccination scenario was projected to prevent as many as 1.9 million (62%) infections in endemic-classified districts (Supplementary Fig. E.4), these areas cover just shy of 10% of the approximately 400 million individuals living in West Africa. However, our model predicts high Lassa fever incidence and disease burden in several ‘non-endemic’ areas. This is consistent with seroprevalence data highlighting extensive underreporting of LASV infection across the region, particularly in Ghana, Côte d’Ivoire, Burkina Faso, Mali, Togo and Benin^14,17–19^. Underreporting of Lassa fever is likely due to a combination of limited surveillance resources in affected countries, the mild and non-specific symptom presentation of most cases, seasonal fluctuations in infection incidence coincident with other febrile illnesses (malaria in particular) and stigma associated with infection, making robust estimation of Lassa fever burden a great challenge^20^. Conversely, low case numbers in some areas estimated to be suitable for transmission^21^ may reflect truly limited burden, driven, in part, by significant spatiotemporal heterogeneity in LASV infection prevalence and the low dispersal rate of *M. natalensis*^22^.

It is important to put Lassa fever’s projected health-economic burden and impacts of vaccination in context, in particular given limited economic resources available for investment in infectious disease prevention in West Africa and, hence, opportunity costs to investing in Lassa vaccination in lieu of other interventions. In Nigeria in 2021, we estimated an annual 48 (95% UI: 19–93) Lassa fever DALYs per 100,000 population. This compares to previous estimates for various emerging, neglected and vaccine-preventable diseases, including trachoma (22 DALYs per 100,000 population in Nigeria in 2019), yellow fever (25), rabies (34), lymphatic filariasis (54), intestinal nematode infections (63), diphtheria (80) and typhoid fever (93)^23^. We further predicted mean TVCs up to $2.20 per dose for preventive campaigns when considering societal costs and monetized DALYs. A global costing analysis across 18 common vaccines estimated a per-dose cost of $2.63 in low-income countries from 2011 to 2020, including supply chain and service delivery costs^24^, suggesting that it may be feasible to achieve a maximum price per dose in line with our TVC estimates. However, real-world costs for any potential forthcoming vaccines are not yet known, and it is important to consider that vaccines currently undergoing clinical trials have distinct dosage regimens^11^ and that our TVC estimates are specific to our model assumptions: a single-dose primary series with a booster dose after 5 years and 10% dose wastage. All else being equal, undiscounted TVC estimates for preventive campaigns would be roughly doubled or reduced by one-third, respectively, for a vaccine not requiring a booster dose or one requiring a two-dose primary series.

The real-world cost-effectiveness of any forthcoming Lassa vaccine will depend not only on its dosage, price and clinical efficacy, estimates of which are not yet available, but also on the alternative interventions available. Novel small-molecule antivirals and monoclonal antibodies are in various stages of development^25,26^ and may represent promising alternatives for prevention of severe Lassa fever. Our results further highlight how the choice of perspective can lead to divergent conclusions regarding vaccine cost-effectiveness^27^. For instance, TVCs were roughly one order of magnitude greater when considering VSL instead of societal costs and monetized DALYs, up to $21.15 from $2.20 per dose. This disparity is consistent with a comparative analysis of health risk valuation, highlighting greatest TVC estimation when using VSL^28^. Although our estimates of vaccine-averted DALYs, societal costs and lost VSL may complement one another to inform priority setting and decision-making^29^, caution is needed when comparing and potentially combining distinct economic metrics (and, hence, perspectives). In particular, the value inherent to VSL may encapsulate both economic productivity and health-related quality of life, so VSL must be considered independently of productivity losses and monetized life-years. Ultimately, defining the full value of vaccination in endemic areas will require ongoing engagement and priority setting across stakeholders^30^ and may benefit from considering broader macroeconomic impacts of vaccination not included in our analysis^31^. However, even if a particular vaccine is identified as a priority by local stakeholders and is predicted to be cost-effective using context-specific willingness-to-pay thresholds and an appropriate perspective, investment will be possible only if vaccination is affordable—that is, if sufficient economic resources are available to cover vaccine program costs.

One major potential benefit to present investment in Lassa vaccination is increased readiness to rapidly develop and deploy vaccines against future LASV variants with pandemic potential. The coronavirus disease 2019 (COVID-19) pandemic demonstrated that prior research on coronaviruses and genetic vaccine technologies gave researchers an important head start on COVID-19 vaccine development in early 2020 (ref. ^32^). In this context, we projected impacts of ambitious vaccination campaigns in response to the emergence of a hypothetical novel LASV variant with pandemic potential. Although it is impossible to predict whether ‘Lassa-X’ will evolve and exactly which characteristics it would have, this modeling represents a plausible scenario for its emergence and spread, totaling, on average, 1.7 million infections, 150,000 hospitalizations and 25,000 deaths over roughly 2 years, resulting in 1.2 million DALYs, $1.1 billion in societal costs and $10.1 billion in VSL lost. We estimate that a vaccine 70% effective against infection and disease, with delivery starting 100 d from the first detected case, could avert roughly one-tenth of Lassa-X’s health-economic burden assuming delivery of approximately 10 million doses per year, or up to three-quarters of its burden given 160 million doses per year. Such ambitious vaccination scenarios are in keeping with the stated goals of the 100 Days Mission^13^, representing an expansive global effort to rapidly respond to emerging pandemic threats. In contrast to LASV, vaccination against Lassa-X was more than three-fold more impactful when blocking infection in addition to disease, due to indirect vaccine protection successfully slowing its explosive outbreak dynamics.

This work has several limitations. First, our projections of Lassa fever burden build upon recent estimates of spillover risk and viral transmissibility but do not account for the potential evolution of these parameters over time, for instance, due to projected impacts of climate change^22^. Second, our model appears to overestimate the magnitude of seasonal fluctuations in incidence, potentially biasing not the total number of infections but, rather, how they are distributed through time. Although peaks in Lassa fever risk during the dry season are well observed, including five-fold greater risk estimated in Nigeria^33^, a large outbreak in Liberia during the rainy season in 2019/2020 highlights that LASV nonetheless circulates year-round^34^. Third, in assuming no LASV seroreversion among previously infected people, our model potentially underestimates the number of infections occurring annually. However, fitting the infection–hospitalization ratio to hospital case data from Nigeria limits the sensitivity of model outcomes to this assumption. Fourth, our evaluation of the economic consequences of Lassa-X is conservative, as we do not account for the exportation of cases outside of West Africa nor potential externalities of such a large epidemic, including negative impacts on tourism and trade, and the oversaturation and potential collapse of healthcare services. Fifth, because poor Lassa fever knowledge has been reported among both healthcare workers and the general population in several endemic areas^35,36^, increased awareness resulting from vaccination campaigns could have positive externalities not considered in our analysis, including the adoption of infection prevention behaviors and timelier care-seeking. Conversely, poor Lassa fever knowledge could limit vaccine uptake, posing challenges to reaching the vaccine coverage targets considered here.

Finally, for both LASV and Lassa-X, we do not stratify risks of infection, hospitalization or death by sex or age, and infections in each country are assumed to be representative of the general population in terms of age, sex, employment and income. Seroepidemiological data from Sierra Leone show no clear association between antibodies to LASV and age, sex or occupation^37^, and studies from hospitalized patients in Sierra Leone and Nigeria show conflicting relationships between age and mortality^3,38,39^. Prospective epidemiological cohort studies, such as the ongoing *Enable* program, will help to better characterize Lassa fever epidemiology—including the spectrum of illness, extent of seroreversion and risk factors for infection and disease—in turn informing future modeling, vaccine trial design and intervention investment^40^. In particular, better quantification of risk in groups thought to be at high risk of infection (for example, healthcare workers) and severe disease (for example, pregnant women) will help to inform targeted vaccination strategies, which are likely to be more cost-effective than the population-wide campaigns considered in our analysis. Nevertheless, a recent stakeholder survey highlights that the preferred vaccination strategy among Lassa fever experts in West Africa is consistent with the vaccine scenarios considered here—that is, mass, proactive campaigns immunizing a wide range of people in high-risk areas—with corresponding demand forecasts reaching up to 100 million doses^41^.

## Conclusion

Our analysis suggests that vaccination campaigns targeting known Lassa fever hotspots will help to alleviate the large health-economic burden caused by this disease. However, expanding vaccination beyond WHO-classified ‘endemic’ districts will be necessary to prevent the large burden of disease estimated to occur in neighboring areas not currently classified as endemic. Improved surveillance is greatly needed to better characterize the epidemiology of Lassa fever across West Africa, helping to inform the design of vaccination campaigns that maximize population health by better targeting those at greatest risk of infection and severe outcomes. In the hypothetical event of a novel, highly pathogenic pandemic variant emerging and devastating the region, our modeling also suggests that the ambitious vaccination targets of the 100 Days Mission could have critical impact, helping to prevent up to three-quarters of associated health-economic burden. The probability of such a variant evolving is exceedingly difficult to predict, but investment in Lassa vaccination now could nonetheless have great additional health-economic value if facilitating a more rapid vaccine response in the event of a pandemic Lassa-related virus emerging.

## Methods

### Inclusion and ethics

This modeling study did not involve the collection or use of any primary individual-level patient data; thus, ethics approval was not necessary. This study included local researchers throughout the research process, including stakeholder meetings, expert feedback and manuscript revision from Lassa fever researchers in regions where Lassa fever is endemic. These researchers are included as co-authors.

### Zoonotic LASV transmission

The incidence of LASV spillover was estimated by extending a previously published geospatial risk model by Basinski et al.^14^ (details in Supplementary Appendix A). In brief, this model synthesizes environmental features, *M. natalensis* occurrence data and LASV seroprevalence data from both rodents and humans to predict rates of zoonotic LASV infection across West Africa. Environmental features were obtained as classification rasters from the Moderate Resolution Imaging Spectroradiometer dataset, including 11 landcover features and seasonally adjusted measures of temperature, rainfall and vegetation. Occurrence data include historical captures of *M. natalensis* confirmed with genetic methods or skull morphology across 167 locations in 13 countries from 1977 to 2017. Rodent seropositivity data cover 13 studies testing *M. natalensis* for LASV across six countries from 1972 to 2014, and human seropositivity data cover 94 community-based serosurveys across five countries from 1970 to 2015.

Consistent with Basinski et al.^14^, we used a GLM to predict human seroprevalence from modeled estimates of spillover risk at the level of 0.05° × 0.05° spatial pixels. To estimate incidence rates, a Susceptible–Infected–Recovered model was used to model transitions among susceptible (seronegative), infected (seropositive) and recovered (seropositive) states. To account for change in human population size over time, this model was augmented with data on per-capita human birth and death rates for each country for each year from 1960 to 2019. Using a forward Euler model with 4-week timesteps, we estimated the number of new infections in each timestep that reproduced modeled seroprevalence estimates in 2015 and stepped this forward to estimate infections in 2019, dividing by the 2019 population size to give the 2019 incidence rate in each pixel^42^. Uncertainty in human LASV seroprevalence from the GLM was propagated forward to generate uncertainty in spillover incidence. Final non-aggregated estimates of spillover incidence from our model (at the pixel level) are shown in Fig. 1, and aggregated estimates at the district level are shown in Supplementary Fig. B.1. Estimates of spillover incidence in endemic districts are shown in Supplementary Figs. B.2 and B.3.

### Human-to-human transmission

We developed a stochastic branching process model to simulate infections arising from human-to-human transmission after spillover infection (Supplementary Appendix C.1). To account for uncertainty in estimated annual spillover incidence, 99 distinct transmission simulations were run, with each one using as inputs a set of LASV spillover estimates corresponding to a particular centile. Each set contains 183 values (one for each district), and the same values are used for each of the 10 years of simulation.

To account for seasonality observed in Lassa fever case reports, annual incidence estimates are distributed across each epidemiological year according to a beta distribution, as considered previously in Lerch et al.^43^. An outbreak tree was generated for each spillover event using an estimate of LASV’s basic reproduction number from the literature (*R*_0_ = 0.063)^43^, estimated from case data from a Lassa fever ward in Kenema Government Hospital, Sierra Leone, from 2010 to 2012 (ref. ^44^). Infections in each outbreak tree are distributed stochastically through time following estimates of LASV’s incubation and infectious periods^43^, and final outbreak trees are combined to generate the daily incidence of human-source infection in each district in the absence of vaccination. See Supplementary Table C.1 for LASV infection and transmission parameters.

### Lassa vaccination campaigns

We included six vaccination scenarios in which limited doses of vaccine are allocated across specific subpopulations of West Africa (see Extended Data Table 2 and Supplementary Appendix C.2 for more details). Vaccine doses are allocated preferentially to populations perceived to be at greatest risk of Lassa fever—that is, those living in districts classified as Lassa fever endemic by the WHO^45^. In some scenarios, a small number of additional doses are allocated to non-endemic districts. In ‘constrained’ scenarios, the total number of vaccine doses is constrained to reflect limited capacity to produce, stockpile and deliver vaccine. For these scenarios, cholera is used as a proxy disease for assumptions relating to vaccine stockpile and target coverage based on recent campaigns in West Africa.

In our vaccination scenarios developed with these constraints in mind, we considered both reactive vaccination (targeting specific districts in response to local outbreaks) and preventive vaccination (mass vaccinating across entire countries or districts regardless of local transmission patterns). Vaccination was assumed to confer immunity for 5 years after a single-dose primary series, with a single-dose booster administered 5 years after the initial dose. Vaccination was applied in the model by ‘pruning’ zoonotic infections and ensuing person-to-person transmission chains—that is, by retrospectively removing infections directly and indirectly averted as a result of vaccination (see Supplementary Appendix C.3 for more details). We did not consider potential side effects of vaccination.

### Health-economic burden of Lassa fever

A decision-analytic model describing the clinical progression of Lassa fever was developed to project the health and economic burden of disease and impacts of vaccination (Supplementary Appendix D.1). Inputs into this model from our spillover risk map and branching process transmission model include, for each year, district and vaccination scenario: the total number of LASV infections, the number of infections averted due to vaccination and the number of infections occurring in vaccinated individuals. The latter is included to account for vaccine preventing progression from infection to disease (Supplementary Appendix D.2). Probability distributions for model parameters were estimated using data from the literature and are described in detail in Supplementary Appendix D.3. In brief, probabilities of hospitalization and death were estimated from reported hospital case data in Edo and Ondo, Nigeria, from 2018 to 2021; durations of illness before and during hospitalization were estimated from a prospective cohort study in a hospital in Ondo from 2018 to 2020; and hospital treatment costs were estimated from patients attending a specialist teaching hospital in Edo from 2015 to 2016 (Supplementary Table D.1)^5,8,38^.

### Model outcomes

Lassa fever health outcomes estimated by our model include mild/moderate symptomatic cases, hospitalized cases, deaths, cases of chronic sequelae (sensorineural hearing loss) after hospital discharge and DALYs. Economic outcomes include direct healthcare costs paid out of pocket or reimbursed by the government, instances of catastrophic or impoverishing out-of-pocket healthcare expenditures, productivity losses, monetized DALYs and the VSL lost (a population-aggregate measure of individuals’ willingness to pay for a reduction in the probability of dying)^46^. We report societal costs as the sum of healthcare costs and productivity losses. All monetary costs are reported in 2021 international dollars ($), and future monetary costs are discounted at 3% per year. Impacts of vaccination are quantified from outputs of the health-economic model as the difference in projected outcomes across parameter-matched runs of the model with and without vaccination.

To calculate the TVC, we first summed relevant monetary costs for each simulation according to the economic perspective considered: healthcare costs and monetized DALYs, societal costs and monetized DALYs or healthcare costs and VSL. The TVC is then calculated as the monetary costs averted due to vaccination divided by the number of vaccine doses allocated, including booster doses and wasted doses, and discounting future vaccine doses at 3% per year.

### Lassa-X

In addition to our analysis of Lassa fever, we consider the emergence of ‘Lassa-X’, a hypothetical future variant of LASV with pandemic potential due to both elevated clinical severity and increased propensity for human-to-human transmission. We assume that the clinical characteristics of Lassa-X are identical to Lassa fever (including sequelae risk and hospital case–fatality ratio), except that Lassa-X is accompanied by a 10-fold increase in risk of hospitalization relative to Lassa fever. Then, to conceive plausible scenarios of Lassa-X transmission informed by empirical data, we assume that the inherent transmissibility of Lassa-X resembles that of Ebola virus during the 2013/2016 West Africa outbreak^47,48^. Ebola virus transmission was chosen as a surrogate for Lassa-X transmission because, like LASV, Ebola virus is a single-stranded RNA virus known to cause outbreaks in West Africa, results in frequent zoonotic spillover to humans from its animal reservoir, causes viral hemorrhagic fever and spreads from human to human primarily through contact with infectious bodily fluids. Based on this conceptualization of Lassa-X, we use a five-step approach to model its emergence and subsequent geospatial spread across West Africa and to estimate the health-economic impacts of reactive ‘100 Days Mission’ vaccination campaigns (described in detail in Supplementary Appendix F).

### Simulation and statistical reporting

For each of 99 runs of the LASV transmission model and 100 runs of the Lassa-X transmission model, health-economic outcomes were calculated via 100 Monte Carlo simulations, in which input parameters for the health-economic model were drawn probabilistically from their distributions (Supplementary Table D.1). In our base case, we assumed that the vaccine is 70% effective only against disease. However, we also included scenarios with vaccine that is 90% effective against disease, 70% effective against both infection and disease and 90% effective against both infection and disease. Final health and economic outcomes, as well as outcomes averted by vaccination, are reported as means and 95% UIs across all simulations over the 10-year time horizon of the model. In sensitivity analysis, we considered a 0% discounting rate, a lower risk of developing chronic sequelae subsequent to hospital discharge and either the same or lower hospitalization risk for Lassa-X relative to LASV. We also conducted a univariate sensitivity analysis to identify the parameters driving outcome uncertainty (see Supplementary Appendix D.4 for more details). Estimates of Lassa fever burden are reported in accordance with the Guidelines for Accurate and Transparent Health Estimates Reporting (GATHER) statement. A GATHER checklist is provided in Supplementary Appendix H.

### Role of the funder

The Coalition for Epidemic Preparedness Innovations (CEPI) commissioned this analysis, and CEPI internal Lassa fever experts were involved in study design by providing knowledge on input parameters and fine-tuning realistic scenarios for vaccine rollout. An earlier version of this work was provided as a report to CEPI.

### Reporting summary

Further information on research design is available in the Nature Portfolio Reporting Summary linked to this article.

## Online content

Any methods, additional references, Nature Portfolio reporting summaries, source data, extended data, supplementary information, acknowledgements, peer review information; details of author contributions and competing interests; and statements of data and code availability are available at 10.1038/s41591-024-03232-y.

## Supplementary information

Supplementary Appendices A–I, Figs. B and D–G and Tables C–E, G and H.

Reporting Summary

## Figures and Tables

**Extended Data Table 1:**
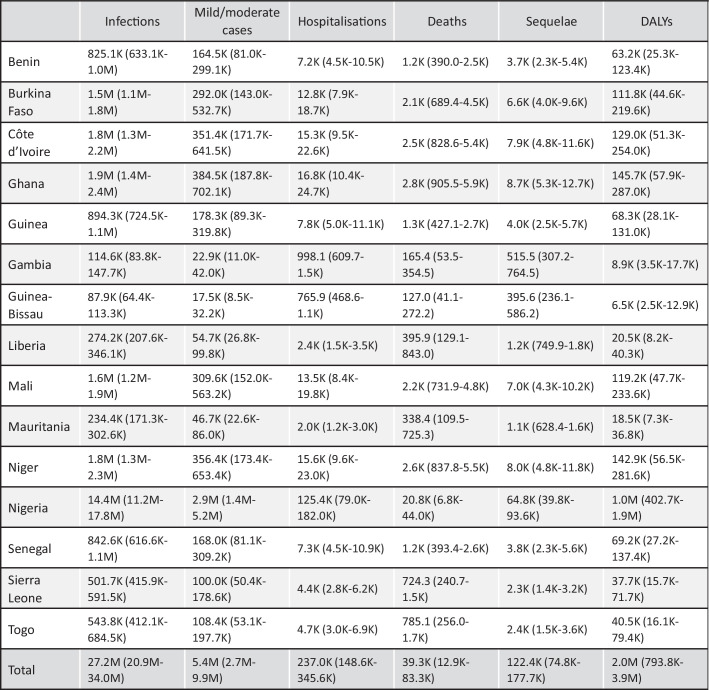
Cumulative health burden of Lassa fever by country over 10 years in the absence of vaccination

All figures represent means (95% UIs) across 100 runs of the health-economic model for each of 99 runs of the infection model for the baseline scenario, assuming a probability of sequelae of 62% among patients discharged from hospital. K, thousand; M, million.

**Extended Data Table 2:**
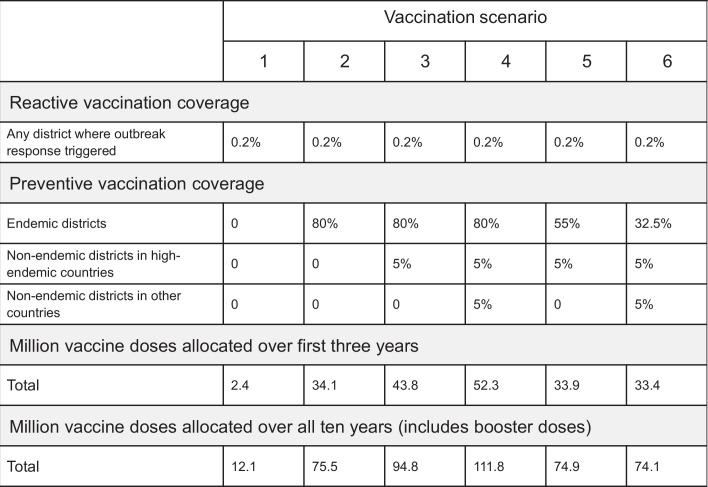
Lassa vaccination scenarios

Scenario 1 includes reactive vaccination only, which is triggered in response to local outbreaks, whereas the remaining scenarios 2–6 include preventive vaccination campaigns in addition to reactive vaccination. Vaccination coverage refers to the percentage of the general population targeted for vaccination in specified districts. Preventive vaccination in scenarios 2–4 is unconstrained—that is, the number of doses reflects desired vaccination coverage levels—whereas preventive vaccination is constrained in scenarios 5 and 6—that is, population coverage is constrained by an upper limit of doses to reflect a limited global vaccine stockpile (Supplementary Table C.2). The small vaccine pool reserved for reactive vaccination (1 million doses annually, shared across all districts proportionately to population size) is available immediately from year 1, whereas vaccination for preventive campaigns is rolled out to different countries in different years, generally to high-endemic, medium-endemic and low-endemic countries in years 1, 2 and 3, respectively (Supplementary Table C.3). A map showing the classification of endemicity across the countries and districts of West Africa is given in Fig. 1. For both reactive and preventive vaccination, booster doses are allocated 5 years after the initial dose, and 90% of available doses are assumed to be delivered (that is, all coverage targets were reduced by 10% wastage).

## Data Availability

The minimum dataset required to run our code and reproduce results is available at 10.5281/zenodo.12751191 (ref. ^49^).

## Code availability

The code underlying our model is available at 10.5281/zenodo.12751191 (ref. ^49^). Data were collected, compiled, analyzed and simulated using R version 4.3.3. Simulations were dispatched using HTCondor version 10.0.4.
