# Supplementary material for: Health and economic impacts of Lassa vaccination campaigns in West Africa
Source: Nat Med. 2024 Aug 28;30(12):3568–77. doi: 10.1038/s41591-024-03232-y (PMC11645265; doi:10.1038/s41591-024-03232-y)
Supplement: Supplementary file 2 — Reporting Summary [file 41591_2024_3232_MOESM2_ESM.pdf]

nature portfolio

Corresponding author(s): David Smith

Last updated by author(s): Jul 3, 2024

Reporting Summary

Nature Portfolio wishes to improve the reproducibility of the work that we publish. This form provides structure for consistency and transparency in reporting. For further information on Nature Portfolio policies, see our Editorial Policies and the Editorial Policy Checklist.

Statistics

For all statistical analyses, confirm that the following items are present in the figure legend, table legend, main text, or Methods section.

n/a	Confirmed	
☐	☒	The exact sample size (*n*) for each experimental group/condition, given as a discrete number and unit of measurement
☒	☐	A statement on whether measurements were taken from distinct samples or whether the same sample was measured repeatedly
☒	☐	The statistical test(s) used AND whether they are one- or two-sided<br>*Only common tests should be described solely by name; describe more complex techniques in the Methods section.*
☐	☒	A description of all covariates tested
☐	☒	A description of any assumptions or corrections, such as tests of normality and adjustment for multiple comparisons
☐	☒	A full description of the statistical parameters including central tendency (e.g. means) or other basic estimates (e.g. regression coefficient) AND variation (e.g. standard deviation) or associated estimates of uncertainty (e.g. confidence intervals)
☒	☐	For null hypothesis testing, the test statistic (e.g. *F*, *t*, *r*) with confidence intervals, effect sizes, degrees of freedom and *P* value noted<br>*Give P values as exact values whenever suitable.*
☒	☐	For Bayesian analysis, information on the choice of priors and Markov chain Monte Carlo settings
☐	☒	For hierarchical and complex designs, identification of the appropriate level for tests and full reporting of outcomes
☒	☐	Estimates of effect sizes (e.g. Cohen's *d*, Pearson's *r*), indicating how they were calculated

Our web collection on statistics for biologists contains articles on many of the points above.

Software and code

Policy information about availability of computer code

Data collection	Data were collected and compiled using R version 4.3.3.
Data analysis	The code underlying our model is available on Github, with a stable release (v.1.0.1) available at https://zenodo.org/doi/10.5281/zenodo.12751191. Data were collected, compiled, analysed and simulated using R version 4.3.3. Simulations were dispatched using HTCondor version 10.0.4.

For manuscripts utilizing custom algorithms or software that are central to the research but not yet described in published literature, software must be made available to editors and reviewers. We strongly encourage code deposition in a community repository (e.g. GitHub). See the Nature Portfolio guidelines for submitting code & software for further information.

Data

Policy information about availability of data

All manuscripts must include a data availability statement. This statement should provide the following information, where applicable:

- Accession codes, unique identifiers, or web links for publicly available datasets
- A description of any restrictions on data availability
- For clinical datasets or third party data, please ensure that the statement adheres to our policy

The minimum dataset required to run our code and reproduce results is available at https://zenodo.org/doi/10.5281/zenodo.12751191.

Research involving human participants, their data, or biological material

Policy information about studies with human participants or human data. See also policy information about sex, gender (identity/presentation), and sexual orientation and race, ethnicity and racism.

Reporting on sex and gender	N/A
Reporting on race, ethnicity, or other socially relevant groupings	N/A
Population characteristics	All population characteristics are aggregated at the national level, including per-capita GNI, employment rate and age
Recruitment	N/A
Ethics oversight	N/A

Note that full information on the approval of the study protocol must also be provided in the manuscript.

Field-specific reporting

Please select the one below that is the best fit for your research. If you are not sure, read the appropriate sections before making your selection.

☒ Life sciences ☐ Behavioural & social sciences ☐ Ecological, evolutionary & environmental sciences

For a reference copy of the document with all sections, see nature.com/documents/nr-reporting-summary-flat.pdf

Life sciences study design

All studies must disclose on these points even when the disclosure is negative.

Sample size	For Lassa fever, we used centiles (n=99) to capture uncertainty in geospatial zoonosis risk and standard samples of n=100 for stochastic runs of our health-economic model, for a sample of n=9,900 in each country (n=15) and year (n=10) across each modelling scenario considered, including vaccination scenarios (baseline + 6 scenarios x 4 levels of vaccine efficacy; n=25), probabilities of sequelae (n=2) and discounting rates (n=2), equaling up to n=148,500,000 data points per model outcome. For Lassa-X, we used standard samples of n=100 for stochastic runs of both our transmission model and of our health-economic model, for a sample of n=10,000 in each country (n=15) across each modelling scenario considered, including vaccination scenarios (baseline + 2 vaccine delays x 3 vaccination rates x 4 levels of vaccine efficacy; n=25), probabilities of sequelae (n=2), severities of Lassa-X (n=3) and discounting rates (n=2), equaling up to n=45,000,000 data points per model outcome.
Data exclusions	No data were excluded.
Replication	Not applicable to simulation modelling.
Randomization	Not applicable to simulation modelling.
Blinding	Not applicable to simulation modelling.

Reporting for specific materials, systems and methods

We require information from authors about some types of materials, experimental systems and methods used in many studies. Here, indicate whether each material, system or method listed is relevant to your study. If you are not sure if a list item applies to your research, read the appropriate section before selecting a response.

Materials & experimental systems

n/a	Involved in the study	
☒	☐	Antibodies
☒	☐	Eukaryotic cell lines
☒	☐	Palaeontology and archaeology
☒	☐	Animals and other organisms
☒	☐	Clinical data
☒	☐	Dual use research of concern
☒	☐	Plants

Methods

n/a	Involved in the study	
☒	☐	ChIP-seq
☒	☐	Flow cytometry
☒	☐	MRI-based neuroimaging

Plants

Seed stocks	N/A
Novel plant genotypes	N/A
Authentication	N/A
